# Prognostic Impact of Longitudinal Monitoring of Radiomic Features in Patients with Advanced Non-Small Cell Lung Cancer

**DOI:** 10.1038/s41598-019-45117-y

**Published:** 2019-06-19

**Authors:** So Hyeon Bak, Hyunjin Park, Insuk Sohn, Seung Hak Lee, Myung-Ju Ahn, Ho Yun Lee

**Affiliations:** 1 Department of Radiology and Center for Imaging Science, Samsung Medical Center, Sungkyunkwan University School of Medicine, Seoul, Korea; 2 Department of Health Sciences and Technology, SAIHST, Sungkyunkwan University, Seoul, Korea; 3 Department of Radiology, Kangwon National University Hospital, Kangwon National University School of Medicine, Chuncheon, Korea; 4 School of Electronic and Electrical Engineering, Sungkyunkwan University, Suwon, Korea; 5 Center for Neuroscience Imaging Research (CNIR), Institute for Basic Science, Suwon, Korea; 6 Statistics and Data Center, Research Institute for Future Medicine, Samsung Medical Center, Seoul, Korea; 7 Department of Electronic Electrical and Computer Engineering, Sungkyunkwan University, Suwon, Korea; 8 Division of Hematology/Oncology, Department of Medicine, Samsung Medical Center, Sungkyunkwan University School of Medicine, Seoul, Korea

**Keywords:** Cancer imaging, Non-small-cell lung cancer

## Abstract

Tumor growth dynamics vary substantially in non-small cell lung cancer (NSCLC). We aimed to develop biomarkers reflecting longitudinal change of radiomic features in NSCLC and evaluate their prognostic power. Fifty-three patients with advanced NSCLC were included. Three primary variables reflecting patterns of longitudinal change were extracted: area under the curve of longitudinal change (AUC1), beta value reflecting slope over time, and AUC2, a value obtained by considering the slope and area over the longitudinal change of features. We constructed models for predicting survival with multivariate cox regression, and identified the performance of these models. AUC2 exhibited an excellent correlation between patterns of longitudinal volume change and a significant difference in overall survival time. Multivariate regression analysis based on cut-off values of radiomic features extracted from baseline CT and AUC2 showed that kurtosis of positive pixel values and surface area from baseline CT, AUC2 of density, skewness of positive pixel values, and entropy at inner portion were associated with overall survival. For the prediction model, the areas under the receiver operating characteristic curve (AUROC) were 0.948 and 0.862 at 1 and 3 years of follow-up, respectively. Longitudinal change of radiomic tumor features may serve as prognostic biomarkers in patients with advanced NSCLC.

## Introduction

Accurate radiological assessment for predicting therapeutic responses and clinical outcomes of non-small cell lung cancer (NSCLC) is important in clinical practice and drug development trials^1^. Cancers have uneven growth patterns, and even within single tumors specific areas may increase in size while other portions remain stable. Intratumoral heterogeneity reflecting heterogeneous biologic phenomenon can affect the rates and patterns of tumor growth^2^. With the development of targeted therapy and immunotherapy, tumor cells remain sensitive to targeted therapy beyond progressive disease (PD) according to Response Evaluation Criteria in Solid Tumors (RECIST), while pseudoprogression occurs in patients undergoing immunotherapy due to infiltration of T cells and macrophages^2,3^. On the other hand, targeted therapy or immunotherapy is used continuously during tumor progression^2,4^, and as assessment of a therapeutic response is usually made between two time points, it may not capture the full spectrum of therapeutic response and could be insufficient in the era of personalized cancer treatment^5^.

Rapid reduction of tumor size is traditionally considered to be associated with better outcomes^6^. Recently, depth of response (DepoR), which indicates the maximum tumor shrinkage from baseline of a target lesion, has begun to be explored as a predictor of long-term treatment outcome^7^. However, recent studies suggest that DepoR should not be used a surrogate of benefit in advanced epidermal growth factor receptor (*EGFR)*-mutant lung cancer^8^. Therefore, parameters such as continuous variables reflecting intratumoral heterogeneity and genomic alterations are needed to assess therapeutic response and clinical outcomes.

Quantitative imaging features may provide comprehensive information on tumor phenotypes and microenvironement^9,10^. Changes in quantitative features are considered a biomarker to accurately assess treatment response and clinical outcomes^11^. Recently, delta-radiomics, a time dependent metric comprised of quantitative features extracted from medical images acquired during the course of treatment has been considered as an emerging biomarker for assessing therapeutic responses and clinical outcomes^12^. We hypothesized that variables capable of reflecting various longitudinal changes in quantitative image features extracted from serial imaging over the course of treatment would be capable of superior prognosis prediction in NSCLC patients. The purpose of this study was to develop biomarkers based on longitudinal changes in quantitative radiomic features in patients with advanced NSCLC, and assess the prognostic power of these biomarkers.

## Results

### Demographics data

This study included 53 patients with stage IV NSCLC (19 men, 34 women; mean age, 58.7 ± 10.6; range, 32–81 years). The histologic types of all patients were adenocarcinoma. Thirty-four patients (64.2%) were never smokers, 62.3% had exon 19 deletion, and 56.6% were treated with EGFR TKIs as a first-line of therapy. Forty (75.5%) patients had metastasis to the central nervous system (CNS), 27 of whom underwent local treatment. Among 53 patients, 27 (50.9%) died. The number of target lesions was 1 in 26 patients (49.0%), 2 in 15 (28.3%), 3 in 10 (18.9%), and 4 in 2 (3.8%). The mean CT follow-up period from baseline to PD was 28 ± 15 months. The characteristics of patients with advanced NSCLCs are presented in Table 1.

**Table 1:** Demographics of 53 patients with advanced NSCLC.

Characteristics	No. of patients (%)
Age*	58.7 ± 10.6 (32–81)
Male:Female	19 (35.8):34 (64.2)
Smoking habits	
Nonsmoker	34 (64.2)
Ex-smoker	11 (20.8)
Current smoker	8 (15.1)
ECOG performance status	
0	2 (3.8)
1	50 (94.3)
2	1 (1.9)
M descriptor	
M1a	9 (17.0)
M1b	44 (83)
Type of EGFR mutation	
Exon 19 deletion	33 (62.3)
L858R	20 (37.7)
Line of EGFR TKIs	
First line	30 (56.6)
Second line	23 (43.4)
EGFR TKIs	
Gefitinib	38 (71.7)
Elrotinib	15 (28.3)
Overall survival	
Death	27 (50.9)
Overall survival	26 (49.1)
Follow-up period (months)*	28 ± 15 (6–54)

Note. ^__^ ECOG, the Eastern Cooperative Oncology group; EGFR, epidermal growth factor receptor; NSCLC, non-small cell lung cancer; TKIs, tyrosine kinase inhibitors.

*Data are mean ± standard deviation and data in parentheses are range.

### Development of radiologic variables reflecting longitudinal volume change

**Figure 1:**
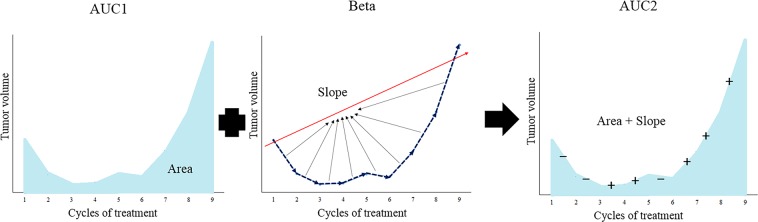
Schematic illustration of the values AUC1, beta and AUC2. AUC1 is defined as the area under the longitudinal change of values. The beta value is the slope calculated by linear regression over time, and represents the slope of the overall longitudinal change. AUC2 is value obtained by considering the slope and area of the longitudinal change. More specifically, subtraction is performed when the slope is negative, and addition is performed when the slope is positive.

**Figure 2:**
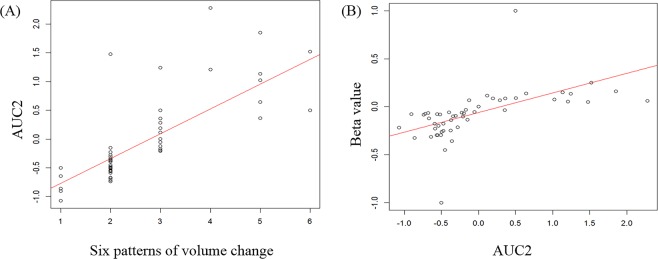
Relationship between AUC2 and curve pattern of volume change (**A**) or beta value (**B**). (**A**) The six patterns of volume changes were follows: (1) reduction only but progressive disease due to nontarget lesions, (2) slow progression after rapid response, (3) rapid progression after rapid response, (4) slow progression after slight reduction, (5) rapid progression after slight reduction, and (6) sequential progression.

AUC1, AUC2, and beta value (Fig. 1) were calculated using the volume change of serial CTs from baseline to PD. The beta value exhibited a good correlation with the six patterns of volume change (r = 0.834, *p* < 0.000). Furthermore, AUC2 reflecting under the area and slope during the follow-up period was well correlated with the six patterns of volume change (r = 0.848, *p* < 0.000) as well as beta value (r = 0.752, *p* < 0.000, Fig. 2). Therefore, AUC2 was considered to be a marker that reflects changes during the follow-up in patients with advanced NSCLC and was used for longitudinal analyses of the 23 quantitative CT features, including volume.

**Figure 3:**
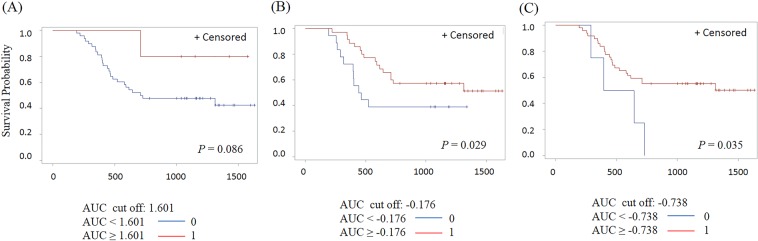
Comparison of Kaplan-Meier curve for overall survival of patients stratified by AUC1 (**A**), beta value (**B**) and AUC2 (**C**).

For the prediction of overall survival time, we calculated the cutoff of AUC1, AUC2, and beta value based on volume. When we selected cutoff values of 1.601 for AUC1, −0.738 for AUC2, and −0.176 for the beta value, the AUC2 and beta values showed significant differences in overall survival time (*p* = 0.035 for AUC2, and *p* = 0.029 for beta value (Fig. 3). However, AUC1 did not exhibit a statistically significant difference with regards to overall survival (*p* = 0.086).

### Prognostic prediction model using longitudinal change of radiomic features

We extracted 23 quantitative features including volume from baseline and follow-up CTs, and calculated the AUC2 of the quantitative CT features extracted from baseline to PD. Based on the cut-off values of quantitative features on baseline CT and AUC2 of quantitative values, all quantitative features were divided into two groups and univariate and multivariate cox regression analyses were performed to identify risk factors to predict overall survival time. Eighteen features were selected in the univariate analysis (supplementary Table 1). In our study, age, sex, the Eastern Cooperative Oncology group (ECOG) performance status, smoking status, type of *EGFR* mutation, CNS metastasis, and local treatment for CNS metastasis were not associated with overall survival time (*p* > 0.05). Six of the 18 features were selected by stepwise selection. Kurtosis of positive pixel values (*p* = < 0.000, HR, 7.992; 95% CI, 2.759–23.148) and surface area (*p* = 0.001, HR, 0.192; 95% CI, 0.075–0.493) on baseline CT, and AUC2 of density (*p* = < 0.000, HR, 0.067; 95% CI, 0.021–0.220), skewness of positive pixel values (*p* = 0.010, HR, 5.142; 95% CI, 1.490–17.747) and entropy at inner portion (*p* = 0.001, HR, 6.196; 95% CI, 2.086–18.406) were associated with overall survival in multivariate cox analysis in patients with advanced NSCLC (Table 2).

**Table 2:** Multivariate Cox regression analyses of overall survival using selected features based on AUC2 and baseline features.

	Selected features	*p* Value	HR	95% CI
AUC2	Density	<0.000	0.067	0.021–0.220
	Skewness of positive pixel value	0.010	5.142	1.490–17.747
	Entropy at inner	0.001	6.196	2.086–18.406
Baseline	M descriptor	0.338	1.806	0.538–6.057
	Kurtosis of positive pixel value	<0.000	7.992	2.759–23.148
	Surface area	0.001	0.192	0.075–0.493

Note. ^__^ CI, confidence interval; HR, hazard ratio.

**Figure 4:**
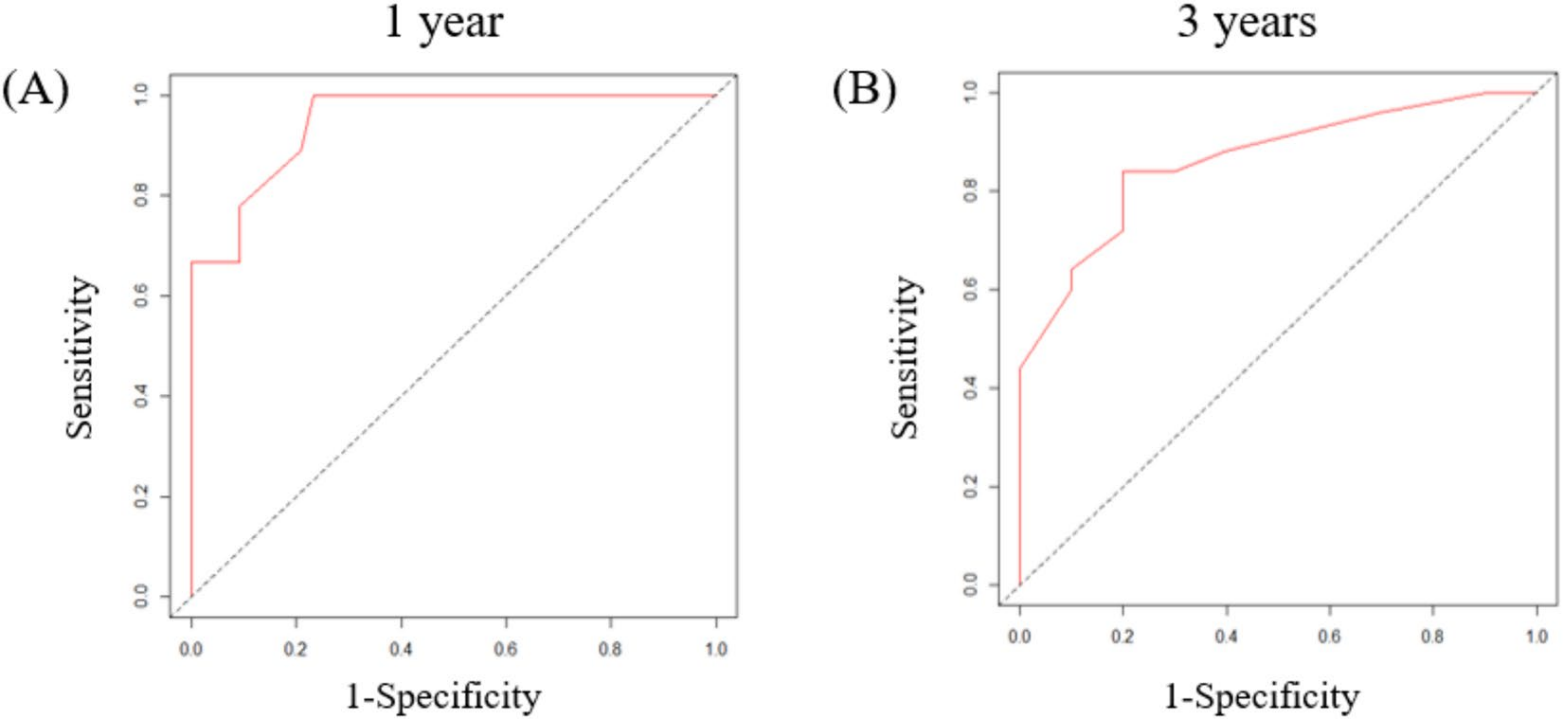
Time-dependent receiver operating characteristic (ROC) curve for prediction model with six features predicting overall survival. The area under the ROC curve (AUROC) was 0.948 at 1 year (**A**), and 0.862 at 3 years (**B**).

The prediction model of overall survival was defined as a linear combination of the five selected features and their regression coefficients. For the prediction model, we constructed a time-dependent ROC curve from which the AUROC was calculated. At 1 and 3 years of follow-up, the AUROCs for overall survival were 0.948, and 0.862 (Fig. 4).

## Discussion

Radiomics studies of NSCLC are usually obtained from images at a single time point. Our studies extracted quantitative features from serial images obtained during the course of treatment in patients with advanced NSCLC. As a result of analyzing longitudinal changes in volume, we identified the variable AUC2 that reflects the slope and area of longitudinal change. Specifically, AUC2 of volume was helpful in predicting overall survival time. Based on this result, we also obtained the AUC2 for 23 quantitative features from serial CT images. Kurtosis of positive pixel values and surface area from baseline CT, AUC2 of density, skewness of positive pixel values, and entropy at inner portion were all associated with overall survival.

Imaging plays a central role in the evaluation of therapeutic response. Decrease in size, vascularity and metabolism, and cavitation within the tumor are well known typical responses to treatment^13^. However, although tumor size can increase with intralesional and/or perilesional hemorrhage, atypical responses consisting of decreased attenuation and metabolism of tumor may also be seen as the tumor becomes homogenous^13^.

As the use of targeted molecular therapy and immunotherapy increases in the era of personalized cancer treatment, morphological changes may be more important than changes in tumor size, and increases in the size of new lesions before dramatic shrinkage may also occur in relation to the immune response^14^. Patients treated with EGFR TKIs can experience a dramatic initial shrinkage of tumor followed by slow progression over several months, a finding that suggests the tumor cells remain sensitive to EGFR TKIs^2,14^. A recent study reported that 88% of patients continued therapy beyond progression according RECIST^15^. Evaluating therapeutic response as a more continuous variable using DepoR has also begun to be explored, and DepoR is used not only in lung cancer, but in other cancers as well including colorectal cancer, breast cancer, and multiple myeloma^16^. A recent study showed that a greater DepoR is associated with better clinical outcome in NSCLC patients receiving an anaplastic lymphoma kinase (ALK) inhibitor or a programed cell death-1 (PD-1) inhibitor^5^. On the other hand, another study reported that DepoR does not correlate with clinical outcome^8^. Therefore, an approach based on a single marker is not sensitive to assessment of therapeutic response due to phenotypic and genomic heterogeneity between lesions, even within an individual tumor^17,18^.

Radiomics is defined as quantitative mapping that converts medial images to automatically extracted quantitative features and provides information related to the comprehensive pathophysiology of a tumor^12,19^. The use of radiomics in lung cancer studies is focused on patient outcomes, nodule discrimination, and assessment of genetic mutations^20^. Whereas most radiomics studies are performed using pretreatment images, the concept of delta-radiomics was introduced to analyze quantitative features from images acquired over the course of treatment by adding a time component to existing radiomic features^12^. Delta-radiomics may be used as indicator to predict response in colorectal liver metastasis, metastatic lung cancer, esophageal cancer, and NSCLC^21–25^. Previous studies have shown that tumor volume is a key predictor reflecting histological tumor aggressiveness and poor prognosis in patients with NSCLC^26,27^. Volumetric measurements that are able to capture tumor changes along the z-axis may be useful for more sensitively and specifically monitoring disease change over time^28–30^. Therefore, in our study, we examined longitudinal changes in tumor volume. We measured additional variables reflecting longitudinal change, and from those variable calculated corresponding AUC1 values reflecting the area of volume change, beta value reflecting slope of change, and AUC2 reflecting area and slope. AUC2 exhibited a good correlation with curve pattern (r = 0.848, *p* < 0.000) and beta value (r = 0.752, *p* < 0.000), whereas the AUC2 of tumor volume was associated with overall survival (*p* = 0.035). Thus, AUC2 may be a new biomarker that reflects longitudinal changes in lesions and predicts overall survival time and treatment response.

Size and volume of a tumor do not reflect changes in tumor heterogeneity and genetic profiles^23^. However, radiomic features can provide more comprehensive information about tumor makeup. A study showed that a novel set of quantitative image features, based on heterogeneities of tumor physiology, was helpful for early prediction of treatment outcome^31^. Therefore, we obtained AUC2 values for 23 quantitative features, including volume, that were previously reported to be associated with lung cancer^32–37^. We examined the association between these 23 pretreatment quantitative features and 23 AUC2s obtained from serial images with overall survival. The result of our study showed that kurtosis of positive pixel values (*p* = <0.000) and surface area (*p* = 0.001) on baseline CT, and AUC2 of density (*p* < 0.000), skewness of positive pixel values (*p* = 0.010), and entropy at inner portion (*p* = 0.001) were all associated with overall survival according to a multivariate analysis in patients with advanced NSCLC.

Targeted therapies developed in recent years can cause tumor necrosis, hemorrhage, or cavitation, and radiomic features based on histograms may be useful for predicting these pathologic response^27^. In patients with a gastrointestinal stromal tumor, treatment with imatinib results in a decrease in tumor density such that the appearance becomes homogenous on CT, even though the decrease in tumor size is minimal at an early posttreatment time point^38^. In our study with advanced NSCLC, density change was associated with increased survival, and it has been suggested that increased homogeneity during the course of treatment is related with therapeutic response and prognosis. Two studies reported that a reduction of entropy reflecting tumor heterogeneity as a parameter to evaluate treatment response in colorectal liver metastases and renal cell carcinoma^24,25^. In our study, we found that the AUC2 of entropy at inner portion was successful for predicting overall survival time in advanced NSCLC. In addition, the AUC2 of the skewness of positive pixel value was found to be predictor of overall survival. Skewness is the distribution pattern of CT attenuation values, and decreased skewness reflects decreased enhancement due to decreased neovascularization during the course of treatment^34^. Therefore, based on our results, changes in tumor heterogeneity were related to overall survival time.

Morphological features reflecting the physical characteristics of tumors are important features related to aggressiveness^18,39^. Irregularity of tumor shape reflecting non-uniform growth of tumors is associated with worse overall survival in patients with lung squamous cell carcinoma and lung adenocarcinoma^32,40^. In our study, surface area on baseline CT was associated with overall survival time in patients with advanced lung cancer, and morphologic features of tumor were considered important predictors related to clinical outcomes.

This study had several limitations. First, the sample size of the present study was small, and our study included patients in various treatment course. However, inclusion of patients with various treatment course may provide a better, more comprehensive assessment of overall survival of patients with advanced lung cancer. A second limitation of this study was that we did not externally validate our results. Therefore, subsequent studies with a large population and external validation are necessary for validation of our results. Third, tumor volume and radiomic features are susceptible to respiration due to collapse and stretching of the tumor and peritumoral lung parenchyma^41,42^. In order to minimize this effect, we excluded patients who had severe respiratory difference in chest CTs or had concurrent disease such as effusion. Fourth, we selected target lesions according to RECIST (version 1.1) criteria. However, current RECIST guidelines may not reflect the overall body tumor load in patients with metastatic cancer. In addition, selection of target lesions is crucial and the result of response classification could be changed by target lesion selection^43,44^. The final limitation of our study was that the ROIs were drawn manually by a single operator. As tumor segmentation is the most critical process, and it is well recognized that interoperator variability of manual segmentation of lesion is high^9^.

## Conclusion

Our study showed that AUC2, a value that reflects the slope and area of longitudinal change calculated from radiomic measurements, is associated with patient survival. Quantitative analysis is a noninvasive technique reflecting comprehensive information such as tumor heterogeneity and genetic profile. In addition, changes in these radiomics features during serial follow-up may provide comprehensive information about response to treatment and prognosis. Therefore, the results of our study suggest that longitudinal change of radiomic tumor features may be useful as prognostic biomarkers in patients with advanced NSCLC.

## Materials and Methods

### Study population

From January 2012 to October 2014, we recruited 80 patients with advanced or recurrent NSCLC with confirmed *EGFR* mutation treated with a tyrosine kinase inhibitor (TKI) and who had been evaluated for therapeutic response on computed tomography (CT) until PD on treatment. Treatment response was assessed according to Response Evaluation Criteria in Solid Tumors (RECIST version 1.1). There was no change in TKI type during the course of treatment. Twenty-seven patients were excluded from our study based on the following exclusion criteria: (1) patients who underwent surgical resection (n = 9), (2) patients without stage IV disease (n = 1), and (3) patient without available serial CTs (n = 17). Thus, a total of 53 patients with advanced NSCLC were included in this retrospective study. The study was approved by the Institutional Review Board (IRB) of Samsung Medical Center (IRB number 2015-10-108), and the requirement for informed consent was waived.

### Image acquisition

All helical CT images were obtained with a 64 detector-row (LightSpeed VCT; GE Healthcare, Waukesha, WI, USA) CT scanner using the following parameters: detector collimation, 1.25 or 0.625 mm; field of view, 36 cm; 125 mA; 120 kVp; beam width, 10–20 mm; beam pitch, 1.375–1.5; section thickness 2.5 mm; and matrix, 512 × 512 mm. All patients underwent chest CT at full inspiration through breath hold to minimize the effect of the tumor motion due to breathing. Chest CT scanning was obtained 90 seconds after the administration of contrast material. A total of 1.5 mL/kg (body weight) Iomeron 300 (Iomeprol, 300 mg iodine/mL; Bracco; Milan, Italy) was injected at an infusion rate of 3 mL/s using a power injector (MCT Plus; Medrad; Pittsburgh, PA, USA). Image data were reconstructed with a soft-tissue algorithm for mediastinal window ranges and a bone algorithm for lung window images. Both mediastinal (width, 300 Hounsfield units [HU]; level, 20 HU) and lung (width, 1500 HU; level −700 HU) window images were displayed for tumor assessment. Chest CT images were obtained every two cycles (8 weeks) during the course of treatment.

### Image analysis

Based on RECIST criteria, up to 5 target lesions on baseline and follow-up chest CT were segmented by drawing a region of interest (ROI) that traced the edge of the lesion on all axial images until the entire lesion was covered. Nontarget lesions were ignored for the analysis of tumor change. Quantitative features were computed over an ROI drawn by a radiologist using MRIcro (version 1.40, Chris Rorden, University of Nottingham, Great Britain). From the baseline to the PD time point, a total of 161 quantitative CT features from each serial CTs were computed using a MATLAB function designed in house (Mathworks Inc., MA, USA). Of the 161 quantitative CT features, 23 features with a known association with lung cancer were selected and used for our analysis (Supplementary Table S2). In all available CTs, volume referred to the measurement of the sum of the target lesions and other quantitative features were measured from the volume weighted average of the target lesions.

We extracted three variables reflecting various patterns of longitudinal change of radiomic features including volume, namely, area under the curve (AUC1), beta value, and AUC2 (Fig. 1). AUC1 is a variable that represents the area of the longitudinal change of a quantitative feature. The beta value refers to the slope calculated by linear regression over time, that is, overall slope of change during follow-up. Third, AUC2 is a value obtained by considering the slope and area of the longitudinal changes of quantitative features. We investigated the relationship between the three variables obtained from the volume of target lesions and six patterns visually divided based on the patterns of longitudinal volume change. The six patterns reflecting longitudinal change of volumes were as follows: 1) reduction only but PD due to non-target lesions, 2) slow progression after rapid response, 3) rapid progression after rapid response, 4) slow progression after slight reduction, 5) rapid progression after slight reduction, 6) and sequential progression.

### Statistical analysis

Analyses were performed using SAS version 9.4 (SAS institute, Cary, NC) and R 3.3.1 (Vienna, Austria; http://www.R-project.org/). A *p* value less than 0.05 was considered statistically significant.

#### Correlation between novel markers and pattern of longitudinal volume change

AUC1, beta value and AUC2 were obtained by the following methods. The regression coefficient of radiomic feature’s value over time was estimated by linear regression and the estimated regression coefficient is denoted as the beta value. The area of radiomic feature from the base line to the next time point was calculated using the formula for the area of a trapezoid. In this way, the area was calculated sequentially up to PD time point. AUC1 represents the sum of all calculated the areas. If the difference of radiomic feature’s values between the current time point and the next time point are positive, add it’s area value, otherwise subtract it’s area value. The total area was calculated by this method, which is denoted as AUC2. The correlations between AUC1, beta value, AUC2 and the six patterns obtained from the longitudinal change of volume were calculated by Spearman’s rank correlation coefficient. The cut-off values that best predicted overall survival time based on AUC1, beta value, and AUC2 of volume were selected as the point with the most significant log-rank *p*-value for all possible cut-off points. Overall survival curves of AUC1, AUC2, and beta value based on cut-off values were estimated using the Kaplan-Meier method.

#### Prognostic prediction model using multivariate analysis

We calculated AUC2 for each of the 23 quantitative features. The cut-off values that best predicted overall survival time for the 23 baseline quantitative features and AUC2 of 23 quantitative features were selected. Univariate and multivariate analyses of the 23 baseline quantitative features with age, sex, ECOG performance state, smoking status, type of EGFR mutation, and AUC2 of the 23 quantitative features were performed using Cox’s proportional hazards model to identify risk factors associated with overall survival. Univariate analysis was used to select 18 features with a *p* value less than 0.05. Spearman’s correlation analysis of 18 features and longitudinal data represented by AUC2 was performed. Some features have a positive or negative correlation. We looked at the variance inflation factor (VIF) to see the collinearity between the features included in the multivariable cox regression. The VIF of all variables was less than and there was no collinearity. To identify significant features on Cox’s proportional hazard model, we considered stepwise selection with Akaike’s Information Criterion (AIC). For significant features of overall survival, a time-dependent receiver operating characteristic (ROC) curve was constructed, and the area under the ROC curve (AUROC) was calculated.

## Supplementary information

Supplementary table
