# Supplementary material for: Prognostic Impact of Longitudinal Monitoring of Radiomic Features in Patients with Advanced Non-Small Cell Lung Cancer
Source: Sci Rep. 2019 Jun 19;9:8730. doi: 10.1038/s41598-019-45117-y (PMC6584670; doi:10.1038/s41598-019-45117-y)
Supplement: Supplementary file 1 — Supplementary table [file 41598_2019_45117_MOESM1_ESM.docx]

**Prognostic Impact of Longitudinal Monitoring of Radiomic Features in Patients with Advanced Non-Small Cell Lung Cancer**

**Running head:** Longitudinal change of radiomic features in lung cancer

So Hyeon Bak, MD, PhD^1,3*^, Hyunjin Park, PhD^4,5*^, Insuk Sohn, PhD ^6*^, Seung Hak Lee, MS^7^, Myung-Ju Ahn, MD, PhD^8^, Ho Yun Lee, MD, PhD^1,2†^

***These authors contributed equally to this study.**

^1^Department of Radiology and Center for Imaging Science, Samsung Medical Center, Sungkyunkwan University School of Medicine, Seoul, Korea

^2^Department of Health Sciences and Technology, SAIHST, Sungkyunkwan University, Seoul, Korea

^3^Department of Radiology, Kangwon National University Hospital, Kangwon National University School of Medicine, Chuncheon, Korea

^4^School of Electronic and Electrical Engineering, Sungkyunkwan University, Suwon, Korea

^5^Center for Neuroscience Imaging Research (CNIR), Institute for Basic Science, Suwon, Korea

^6^Statistics and Data Center, Research Institute for Future Medicine, Samsung Medical Center, Seoul, Korea

^7^Department of Electronic Electrical and Computer Engineering, Sungkyunkwan University, Suwon, Korea

^8^Division of Hematology/Oncology, Department of Medicine, Samsung Medical Center, Sungkyunkwan University School of Medicine, Seoul, Korea

***Corresponding Author:**

Ho Yun Lee, MD, PhD

Department of Radiology and Center for Imaging Science, Samsung Medical Center, Sungkyunkwan University School of Medicine, 81 Irwon-Ro, Gangnam-Gu, Seoul 06351, Korea; and Department of Health Sciences and Technology, SAIHST, Sungkyunkwan University, Seoul 06351, Korea

Tel) 822-3410-2502

Fax) 822-3410-0049

E-mail) [hoyunlee96@gmail.com](mailto:hoyunlee96@gmail.com)

Supplement Table S1. Prediction of the survival based on AUC2 and baseline features

|  | **Selected features** | ***p* Value** | **HR** | **95% CI** |
| --- | --- | --- | --- | --- |
| AUC2 | Volume | 0.048 | 0.295 | 0.088-0.988 |
|  | Density | 0.006 | 0.271 | 0.107-0.687 |
|  | Mass | 0.041 | 0.282 | 0.084-0.949 |
|  | Skewness of positive pixel value | 0.043 | 2.774 | 1.033-7.447 |
|  | Skewness at inner | 0.029 | 2.758 | 1.110-6.851 |
|  | Skewness at delta | 0.019 | 0.276 | 0.094-0.808 |
|  | Kurtosis of positive pixel value | 0.029 | 0.380 | 0.159-0.907 |
|  | Kurtosis at outer | 0.040 | 0.280 | 0.083-0.945 |
|  | Entropy at inner | 0.040 | 2.772 | 1.048-7.333 |
| Baseline | Age | 0.570 | 1.011 | 0.973-1.052 |
|  | Sex | 0.151 | 1.893 | 0.792-4.528 |
|  | ECOG | 0.997 | >999.999 | <0.001 - >999.999 |
|  | Smoking | 0.514 | 1.320 | 0.573-3.038 |
|  | Type of *EGFR* mutation | 0.316 | 0.653 | 0.283-1.503 |
|  | M descriptor | 0.626 | 1.303 | 0.449-3.785 |
|  | CNS metastasis | 0.465 | 0.710 | 0.283-1.780 |
|  | Local treatment for CNS metastasis | 0.179 | 0.583 | 0.265-1.281 |
|  | Volume | 0.006 | 0.020 | 0.001-0.317 |
|  | Mass | 0.006 | 0.020 | 0.001-0.317 |
|  | Skewness at outer | 0.011 | 0.184 | 0.050-0.674 |
|  | Kurtosis of whole pixel value | 0.015 | 0.061 | 0.006-0.583 |
|  | Kurtosis of positive pixel value | 0.044 | 2.219 | 1.024-4.809 |
|  | Kurtosis at inner | 0.038 | 0.103 | 0.012-0.883 |
|  | Entropy of whole pixel value | 0.001 | 17.154 | 3.295-89.316 |
|  | Entropy at outer | 0.001 | 17.154 | 3.295-89.316 |
|  | Surface area | 0.001 | 0.208 | 0.083-0.521 |

Data are features selected as variables using univariate cox regression analysis

Abbreviations: CNS, central nervous system; ECOG, the Eastern Cooperative Oncology group; CI, confidence interval; EGFR, epidermal growth factor receptor; HR, hazard ratio

Supplement Table S2. Twenty-three quantitative CT features based on histogram, shape and size

| **Histogram features** | | **Shape and size features** |
| --- | --- | --- |
| **Whole pixel value** | **Positive pixel value** |  |
| Energy | Energy | Volume |
| Skewness* | Skewness | Density |
| Kurtosis* | Kurtosis | Mass |
| Entropy* | Entropy | Spherical disproportion |
| Uniformity |  | Surface area |
| HU at the 75th percentile |  |  |

* These features are calculated form whole pixels and ROI sub-sampling (inner 2/3, outer 1/3, delta)

Abbreviations: CT, computed tomography; ROI, region of interest
